# Association between serum low‐density neutrophils and acute‐onset and recurrent Guillain–Barré syndrome

**DOI:** 10.1002/brb3.2456

**Published:** 2021-12-10

**Authors:** Kaixi Ren, Aili Yang, Jiarui Lu, Daidi Zhao, Miao Bai, Jiaqi Ding, Tiaoxia Wei, Hongzeng Li, Jun Guo

**Affiliations:** ^1^ Department of Neurology Tangdu Hospital Fourth Military Medical University Xi'an China; ^2^ Department of Endocrinology Tangdu Hospital Fourth Military Medical University Xi'an China; ^3^ State Key Laboratory of Cancer Biology, Department of Medical Genetics and Developmental Biology Fourth Military Medical University Xi'an China

**Keywords:** acute‐onset, Guillain–Barré syndrome, low‐density neutrophils, prognostic indicator, recurrence

## Abstract

**Background and Aim:**

Guillain–Barré syndrome (GBS) is one of the most common causes of acute flaccid paralysis. A timely assessment of this disease condition and its treatments are of vital importance to patients diagnosed with GBS. The purpose of this study is to investigate the variation trend of neutrophils along with disease courses and assess the prognostic value of serum low‐density neutrophils (LDNs) in the acute‐onset and recurrence of GBS.

**Methods:**

A total of 176 GBS patients were recruited. Patients were evaluated with Medical Research Council (MRC) sum score and the Hughes Functional Grading Scale score upon admission. Peripheral blood samples were collected for routine testing. Flow cytometry analysis was performed to identify LDNs. All patients were followed up to collect disease condition data.

**Results:**

The total neutrophil ratios and counts were significantly higher in patients with acute‐onset GBS compared to healthy controls (HCs). These counts/ratios decreased during remission and re‐elevated in recurrent GBS patients. However, no correlation was observed between the total neutrophil counts/ratios and the MRC sum score. The LDNs collected from different GBS courses were identified using flow cytometry. The counts and ratios were significantly higher in acute‐onset GBS and recurrent GBS compared to HCs and patients in remission. The LDN counts/ratios displayed a negative correlation with the MRC sum scores in acute‐onset GBS and recurrent GBS.

**Conclusion:**

Our findings suggest that LDN counts/ratios are positively correlated with the acute‐onset and recurrence of GBS and its severity. Therefore, LDNs might serve as an accessible prognostic indicator for disease progression monitoring.

## Graphical abstract

The LDNs counts/ratios were significantly higher in patients with acute‐onset GBS. The LDN counts/ratios displayed a negative correlation with the MRC sum scores in acute‐onset GBS.

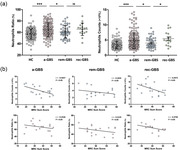

## 1 INTRODUCTION

Guillain–Barré syndrome (GBS) is one of the most common causes of acute flaccid paralysis. It is characterized by acute onset with preceding infection, limb weakness, areflexia, and cerebrospinal fluid albuminocytological dissociation (Asbury & Cornblath, 1990; Carmona‐Rivera & Kaplan, 2013; Casserly et al., 2017). Timely assessment of this disease condition is of vital importance during the acute phase for treatment decisions and prognostic evaluations. Although GBS is often characterized by self‐limiting and monophasic disease courses, recurrent cases are noticed in 5%−10% of patients (Cloke et al., 2012; Coffelt et al., 2015). These patients experience multiple periods of deterioration. However, to date, there is a lack of promising indicators that guide the initiation of treatment for GBS until disease progression. Thus, identifying prognostic factors to determine the phase of acute onset and recurrence of GBS is essential for clinical decisions and disease recovery.

Neutrophils play significant roles in both acute and chronic inflammation by regulating the inflammatory process through cytokine secretion, immune cell recruitment, microbe phagocytosis, and antimicrobial molecule degranulation (Denny et al., 2010). Increasing evidence suggests that neutrophils are heterogeneous in morphology and function (Drifte et al., 2013). Low‐density neutrophils (LDNs), initially observed by density gradient isolation, have recently been recognized as a subtype of neutrophils with particular features (Fortunati et al., 2009). It has been reported that LDNs expressing CD15^+^CD11b^+^CD33^+^HLA‐DR^−^ are activated neutrophils that undergo degranulation and a series of inflammatory processes in multiple autoimmune diseases such as systemic lupus erythematosus (SLE), psoriasis, and arthritis (Fujimi et al., 2012; Goodfellow & Willison, 2016; Grand'Maison et al., 1992; Grayson et al., 2015). Also, a series of nerve system demyelinating autoimmune diseases, for instance, neuromyelitis optica spectrum disorder (NMOSD), multiple sclerosis (MS), and autoimmune encephalitis have been studied to have relevance to neutrophils (Hacbarth & Kajdacsy‐Balla, 1986; Hassani et al., 2020; Hoffmann et al., 2013; Huang et al., 2018). However, the association between LDNs and the acute onset and recurrence of GBS remains unclear.

The present study aimed to analyze the variation trend of neutrophils along with disease courses and assess the prognostic value of LDNs in the acute onset and recurrence of GBS.

## 2 METHODS AND METHODS

### 2.1 Patients

We examined 187 patients diagnosed with GBS between July 2014 and July 2019 at Tangdu Hospital, China. Clinical evaluations were performed immediately upon admission. Routine blood tests were performed for all inpatients. These patients were diagnosed as classical GBS subtypes based on the National Institute of Neurological Disorders and Stroke criteria (NINCDS) (Hüner et al., 2018), including acute inflammatory demyelinating polyneuropathy (AIDP), acute motor axonal neuropathy (AMAN), and acute motor and sensory axonal neuropathy (AMSAN). Clinical diagnosis was supported by symptoms of acute flaccid paralysis, progressive phase period, nerve conduction velocity tests (Inoue et al., 2003), and cerebrospinal fluid albuminocytological dissociation. The eligibility criteria included age group of > 15 years of both gender, patients with progressive weakness in limbs and areflexia in weak limbs. Symptomatic progressions were described as definite neurological aggravation compared with the patient's condition the day before. Chronic inflammatory demyelinating polyradiculoneuropathy (CIDP) and variant subtypes of GBS, like Miller–Fisher syndrome patients, were excluded from the present study. Also, we excluded 11 patients with coexisting diseases that might have influenced the evaluation: diabetes (*n* = 7), confirmed cancer (*n* = 3), and systemic vasculitis (*n* = 1). A total of 176 patients were included in the study and evaluated using the Medical Research Council (MRC) sum score and Hughes functional grading scale score (Kieseier et al., 2018). Briefly, the MRC sum score is used to evaluate six groups of muscles including proximal and distal upper limbs and lower limbs on both sides, with scores ranging from 0 to 60. The individual muscle group is scored based on myodynamia from 0 to 5: 0, no visible contraction; 1, visible contraction without movement; 2, active movement of the limb, but not against gravity; 3, active movement against gravity over (almost) the full range; 4, active movement against gravity and resistance; and 5, normal power. Hughes functional grading scale score ranges from 1 to 6: 1, minor symptoms and capable of running; 2, able to walk 10 m or more without assistance but unable to run; 3, able to walk 10 m across an open space with help; 4, bedridden or chairbound; 5, requiring assisted ventilation for at least part of the day; and 6, dead. In addition, age, sex, infection history, sensory deficits, cranial nerve involvement, and acute treatment (intravenous immunoglobulins [IVIgs] or plasma exchanges [PEs]) were recorded. In addition, 144 healthy donors were recruited for the blood tests. This study was approved by the Ethics Committee of Tangdu Hospital (TDLL‐KY‐202106‐02). All patients and healthy donors provided written informed consent to participate in the study.

### 2.2 Routine blood tests and flow cytometry

Peripheral blood samples were collected for routine blood tests and identified through flow cytometry during the first hospitalization, clinical remission, and disease recurrence. Briefly, blood was freshly collected into anticoagulant tubes from patients and healthy donors on experimental days. As for flow cytometry analysis, blood was added into red blood cells lysis buffer immediately after collection. After the process of red blood cells lysis, cells were centrifuged to collect the remaining white blood cells for further cell counting and fluorescent‐conjugated antibodies staining. All procedures were performed in a dark room. 7‐aminoactinomycin D (7AAD) was added in the resuspension solutions before tests to eliminate dead cells. Cells were never allowed to be frozen or kept overnight before FACS analysis. FITC anti‐HLA DR (#307604), Pacific Blue anti‐CD11b (#101224), PE‐Cy7 CD15 (#323030), and APC‐Cy7 CD33 (#366614) were purchased from BioLegend (San Diego, CA, USA). Flow cytometry analysis was carried with an FACSCanto flow cytometer (BD Immunocytometry Systems, San Jose, CA, USA).

### 2.3 Statistical analyses

Statistical analyses were performed by GraphPad Prism software (7.0 version). Categorical data are presented as numbers and percentages, and quantitative data are presented as the mean ± standard error of the mean. Intergroup differences were assessed by one‐way analysis of variance followed by Bonferroni post hoc test, and Pearson's correlation analysis was performed to examine the association between the MRC sum score and total neutrophil ratios/counts and LDN ratios/counts. *p* < .05 was considered statistically significant.

## 3 RESULTS

### 3.1 Patient characteristics

We recruited 176 GBS patients who met the diagnostic criteria for AIDP and AMAN. The incidence rate and recurrence rate were similar among all age groups. A total of 83 (47.2%) patients reported having a history of infection. The patients with Hughes functional grading scale score ≥ 4 accounted for 35.8% and 52.9% in acute‐onset GBS and recurrent GBS patients, respectively. Sensory deficits were observed in 64 (36.4%) patients. Thirteen (7.39%) of the total patients reported cranial nerve involvement. The proportion of patients who received IVIgs or PEs was 92.6%. We followed up with all patients for at least 12 months since disease onset. After hospital treatment and discharge, 15 patients reported GBS recurrence within 3 months, and two of them reported two recurrences (Table 1). Due to lack of compliance after improvement or economic reasons, only 51 improved patients returned to the hospital for re‐examination as requested.

**TABLE 1:** Characteristics of the patients (*n* = 176)

Variables	Acute‐onset GBS *n* (%)	Recurrent GBS *n* (%)
Total	176	17
GBS subtypes		
AIDP	96 (54.5%)	9 (52.9%)
Axonal GBS (AMAN/AMSAN)	65 (37.0%)	6 (35.3%)
Equivocal	15 (8.5%)	2 (11.8%)
Age (years)		
> 60	35 (19.9%)	3 (17.6%)
41−60	116 (65.9%)	11 (64.7%)
≤ 40	25 (14.2%)	3 (17.7%)
Female/male	104/72 (59.1%/40.9%)	12/5 (70.6%/29.4%)
Symptoms preceding infection		
Diarrhea	45 (25.6%)	7 (41.2%)
Upper respiratory tract infection	38 (21.6%)	10 (58.8%)
NCV findings		
Demyelinated	96 (54.6%)	9 (52.9%)
Axonal	65 (37.0%)	5 (29.4%)
Equivocal	10 (5.70%)	3 (17.7%)
Normal	5 (2.80%)	0 (0.00%)
Hughes functional grading scale score		
1	18 (10.2%)	0 (0.00%)
2	32 (18.2%)	2 (11.8%)
3	63 (35.8%)	6 (35.3%)
4	53 (30.1%)	8 (47.1%)
5	10 (5.70%)	1 (5.80%)
MRC sum score		
60–51	24 (13.6%)	2 (11.8%)
50–41	45 (25.6%)	5 (29.4%)
40–31	65 (36.9%)	8 (47.0%)
30–21	23 (13.1%)	2 (11.8%)
20–0	19 (10.8%)	0 (0.00%)
Sensory deficits	64 (36.4%)	4 (23.5%)
Cranial nerve involvement	13 (7.39%)	2 (11.8%)
Acute phase treatment (IVIg/PE)	163 (92.6%)	17 (100%)

Abbreviations: AIDP, acute inflammatory demyelinating polyneuropathy; AMAN, acute motor axonal neuropathy; AMSAN, acute motor and sensory axonal neuropathy; GBS, Guillain–Barré Syndrome; IVIg, intravenous immunoglobulins; NCV, nerve conduction velocity; PE, plasma exchange.

### 3.2 Variation of total neutrophils in the whole Guillain–Barré syndrome course

**FIGURE 1:**
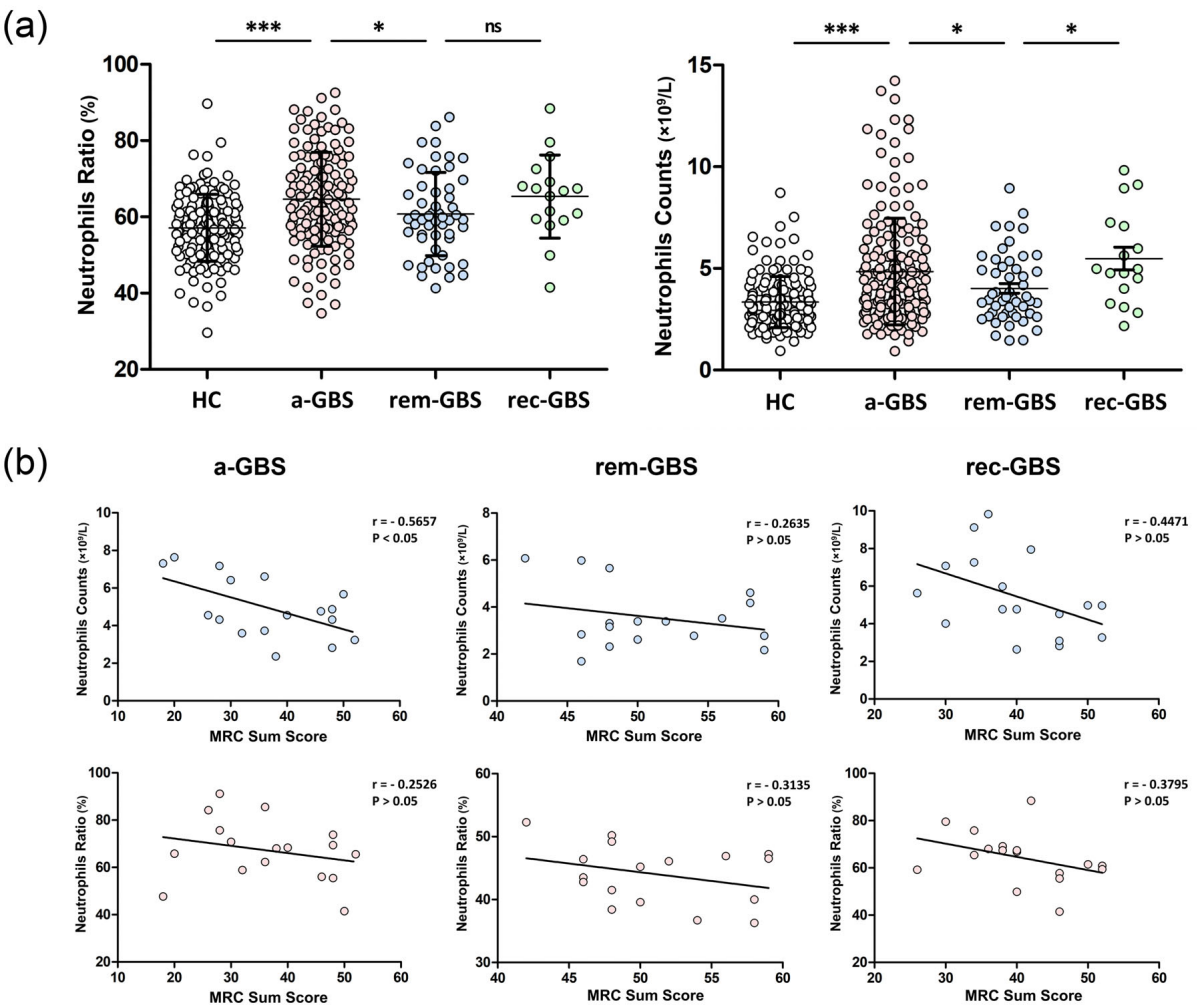
(a) The variation of total neutrophil ratios and counts during different GBS courses. (b) Association between total neutrophils and disease severity. Pearson's correlation was used to analyze the relevance of total neutrophil counts (upper) and total neutrophil ratio (lower) to the Medical Research Council sum score. HCs, healthy controls; GBS, Guillain–Barré syndrome; a‐GBS, acute‐onset GBS; rem‐GBS, GBS in remission; rec‐GBS, recurrent GBS (^*^ *p* < .05, ^***^ *p* < .001)

The total neutrophil ratios and counts were acquired by routine blood tests from healthy controls (HCs), GBS patients with acute‐onset GBS, GBS patients in remission, and recurrent GBS patients. As shown in Figure 1a, neutrophil ratios (64.6 ± 1.05%) and counts (4.84 ± 0.20 × 10E9/L) were significantly elevated in acute‐onset GBS patients compared with HCs (57.1 ± 0.73%, 3.34 ± 0.11 × 10E9/L) and decreased in remission patients along with improved symptoms (60.7 ± 1.49%, 4.02 ± 0.24 × 10E9/L). However, in recurrent GBS patients, neutrophil ratios and counts increased again (65.3 ± 2.64%, 5.48 ± 0.55 × 10E9/L).

### 3.3 Association between total neutrophils and disease severity

The Pearson correlation was calculated to evaluate the association between total neutrophil counts/ratios and patient MRC sum score, reflecting limb myodynamia. We randomly selected the same number of acute‐onset GBS and remission patients as recurrent GBS patients (*n* = 17). As shown in Figure 1b, neutrophil counts in the acute‐onset GBS group were negatively correlated with their MRC sum scores (*r* = −0.5657, *p* = .02). However, the neutrophil ratios in the acute‐onset GBS group showed no correlation with the score. There were no significant correlations between neutrophil counts/ratios and MRC sum score in the other groups.

### 3.4 Identification of low‐density neutrophils and their variation in the whole Guillain–Barré syndrome course

**FIGURE 2:**
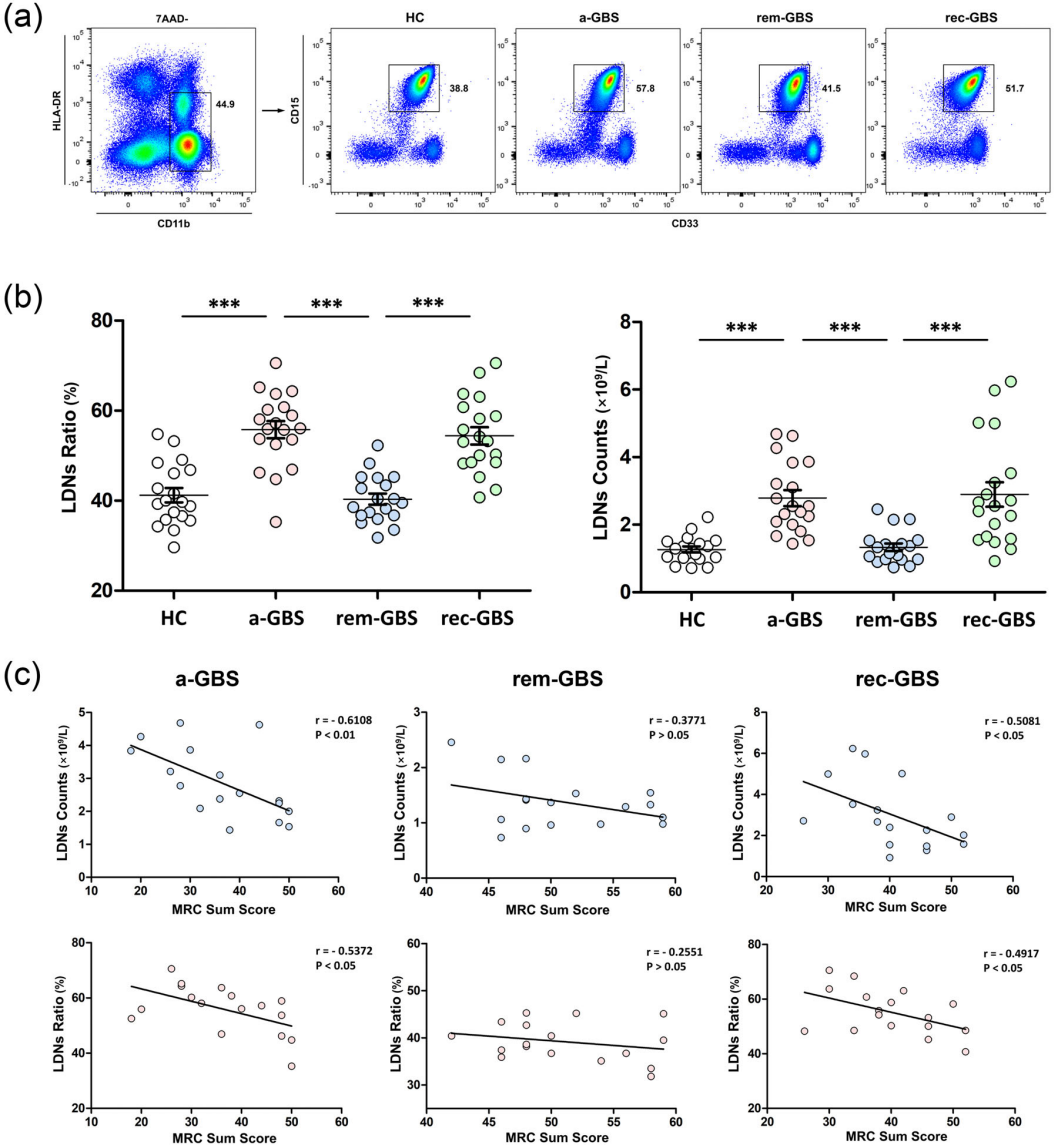
(a) Flow cytometry was used for gating HLA‐DR‐CD11b+CD15+ CD33+ LDNs. Data were shown as proportions of LDNs in whole blood cells. (b) Variation of LDN ratios and counts during different GBS courses. (c) Association between low‐density neutrophils (LDNs) and disease severity. Pearson's correlation was used to analyze the relevance of LDN counts (upper) and LDN ratios (lower) to the Medical Research Council sum score (^***^ *p* < .001)

Since LDNs play essential roles in autoimmune disease‐derived inflammation, flow cytometry was used for further analysis. LDNs displayed immature and activated characteristics with the expression of CD15^+^CD11b^+^CD33^+^HLA‐DR^−^. Live and antibody‐labeled cells were gated and counted (Figure 2a). LDNs accounted for 41.2 ± 1.6%, 55.8 ± 1.9%, 40.3 ± 1.2%, and 54.4 ± 1.9% of peripheral blood cells in the HC, acute‐onset GBS, GBS in remission, and recurrent GBS groups, respectively. The cell counts were 1.27 ± 0.09 × 10E9/L, 2.79 ± 0.24 × 10E9/L, 1.33 ± 0.11 × 10E9/L, and 2.89 ± 0.36 × 10E9/L in the above four groups, respectively. The LDN ratios and counts were significantly higher in the acute‐onset GBS and recurrent GBS groups compared to the HCs and remission group (Figure 2b).

### 3.5 Association between low‐density neutrophils and disease severity

We re‐evaluated the association between LDN counts/ratios and MRC sum score in the same group of patients. During the acute phase of GBS, LDN counts/ratios were negatively correlated with MRC sum scores (counts: *r* = −0.6108, *p* = .009; ratio: *r* = −0.5372, *p* = .02). During remission, the correlation between LDN counts/ratios and MRC sum score was not as significant as that between total neutrophil counts/ratios and MRC sum score (counts: *r* = −0.3771, *p* = .13; ratio: *r* = −0.2551, *p* = .32). However, when recurrence occurred, LDN counts/ratios displayed a negative correlation with MRC sum scores (counts: *r* = −0.5081, *p* = .03; ratio: *r* = −0.4917, *p* = .04) (Figure 2c). This indicates the essential role of LDNs in immune‐mediated neuropathy compared to the correlation results of total neutrophils.

## 4 DISCUSSION

The results of our study showed that LDN ratios and counts in the peripheral blood might serve as potential prognostic indicators in both acute onset and recurrent phases of GBS. Neutrophils play essential roles in the initiation or regulation of local or systemic immune responses, and their hyperactivation is associated with continuous inflammation and tissue damage. Their functions in either GBS or other immune‐mediated diseases have been described in previous studies (Hacbarth & Kajdacsy‐Balla, 1986; Hassani et al., 2020; Hoffmann et al., 2013; Huang et al., 2018). For instance, neutrophil‐to‐lymphocyte ratio (NLR) is regarded as a biomarker indicating a GBS pathophysiological or clinical status (Kolaczkowska & Kubes, 2013; Leonhard et al., 2019; Lin et al., 2011). An increased NLR was observed in GBS patients, and a decrease was observed with IVIg treatment. In our study, the neutrophil ratios and counts were elevated in the acute/recurrent phase and maintained at low levels in the remission phase and in healthy donors. The MRC sum score was used for the comprehensive assessment of patients’ physical status. Neutrophil counts were negatively correlated with MRC sum scores in the acute‐onset GBS group, which might indicate that elevation of neutrophils counts leads to worse disease conditions. However, there were no significant correlations between neutrophil counts/ratios and MRC sum scores in the other groups. Although neutrophil counts were significantly higher in recurrent GBS compared to GBS in remission, there was a lack of prognostic value for disease conditions.

Recent studies on neutrophil heterogeneity have identified several subtypes of neutrophils with distinct characteristics. LDNs, initially described as low buoyant density neutrophils (Malide et al., 1995), are considered immature, activated, and degranulated cells with immunomodulatory capabilities (Morisaki et al., 1992; Ng et al., 2019; Ozdemir, 2016). They show increased reactivity to chemotactic factors and inhibitory effects on T cell proliferation and natural killer cell activation (Piatek et al., 2018; Rahman et al., 2019). They are also characterized by decreased phagocytic capacity and impaired reactive oxygen species production (Rosales, 2020). Inflammatory processes, such as degranulation, might contribute to buoyant density loss. It has been reported that LDNs secrete higher levels of pro‐inflammatory cytokines, including interleukin 8 (IL‐8), IL‐6, type I interferons, and tumor necrosis factor‐α (Ruts et al., 2010; Saeed et al., 2019). Flow cytometry was used for analysis based on the cell surface markers. LDNs expressed low CD14 but high CD15, CD11b, and CD33, with a lack of major histocompatibility complex class II (Ruts et al., 2010; Sagiv et al., 2015). To date, LDNs have been reported in many immune‐mediated diseases such as SLE, psoriasis, vasculitis, sepsis, human immunodeficiency virus infection, and pristane‐induced arthritis (Grayson et al., 2015; Hacbarth & Kajdacsy‐Balla, 1986; Shahrizaila et al., 2021; Spiegel et al., 2016; Tay et al., 2020). LDNs are regarded as potentially pathogenic cell types or prognostic indicators throughout the above‐mentioned disease courses. However, in GBS, which is previously described as an adaptive immune disease, neutrophils detections are not valued as B lymphocytes or antibodies. In our study, we detected LDNs in different clinical courses of GBS patients for the first time and observed the variation tendency of LDNs in acute‐onset and recurrent phases of GBS in particular. After cell gating with a certain flow cytometry strategy, both LDN ratios and counts showed significant differences along with changes in disease conditions. They increased in acute‐onset GBS and decreased to basal levels in the remission phase. In recurrent GBS, the LDN counts and ratios were again elevated significantly. Further assessment of the associations between LDNs and MRC sum scores of patients in different phases indicated that LDNs were specifically correlated with disease recurrence. As the number of LDNs increased, patients experienced clinical deterioration in symptoms and signs. Immediate IVIg retreatment was administered to these patients, and improvements were observed (not shown).

The indications for GBS recurrence have remained controversial worldwide. For instance, autoimmune anti‐ganglioside antibodies are frequently detected for disease assessment and prognosis, since GBS is generally recognized as a humoral immune‐mediated disease. However, the antibodies might not be as vital as expected. They were neither used as alternative diagnostic criteria nor regarded as indicators of clinical fluctuations (Uncini & Kuwabara, 2012; van Koningsveld et al., 2007; Wang et al., 2018). The initiating factors or other underlying mechanisms of GBS recurrence remain unclear, complicating prognostic estimation. Our findings showed that, as an accessible immunological indicator, LDN variation might be correlated with GBS progression and disease severity in different phases. However, larger sample sizes and extended follow‐up time are needed for further clinical studies, since the number of recurrent patients is small in the present study. Additional studies focused on treatment influence and LDNs modulatory mechanisms are yet to be conducted.

## CONFLICT OF INTEREST

The authors declare no conflicts of interest.

### PEER REVIEW

The peer review history for this article is available at https://publons.com/publon/10.1002/brb3.2456

## Data Availability

All data included in this study are available upon request by contact with the corresponding author.
